# Health-related quality of life as an endpoint in oncology phase I trials: a systematic review

**DOI:** 10.1186/s12885-019-5579-3

**Published:** 2019-04-16

**Authors:** Frédéric Fiteni, Isabelle Le Ray, Ahmad Ousmen, Nicolas Isambert, Amélie Anota, Franck Bonnetain

**Affiliations:** 1 Department of Medical Oncology, University Hospital of Nîmes, Rue du Pr Henri Pujol, 30029 Nîmes Cedex 9, France; 2 Institut de Recherche en Cancérologie de Montpellier (IRCM), INSERM U1194, Montpellier, France; 3 University of Montpellier, Montpellier, France; 4 Department of Neonatology, Strasbourg University Hospital, Strasbourg, France; 5 Department of Medical Epidemiology and Biostatistics, Karolinska Universitet, Stockholm, Sweden; 6 Methodology and Quality of Life in Oncology Unit, INSERM UMR 1098, University Hospital of Besançon, Besançon, France; 7 Medical Oncology Department, Centre GF Leclerq, Dijon, France; 8 French National Platform Quality of Life and Cancer, Dijon, France

**Keywords:** Health-related quality of life, Phase I trial, Endpoint, Recommended phase II dose

## Abstract

**Background:**

Phase I trials aim to identify the recommended dose for further development. Health-related quality of life (HRQoL) could be a complement to the usual National Cancer Institute Common Terminology Criteria for Adverse Events (NCI-CTCAE) scale to detect adverse events and define the doses. The objective of this study is to review the phase I in oncology which used HRQoL as endpoint.

**Methods:**

A search in PubMed database identified phase I trials in oncology with HRQoL as endpoint, published between January 2012 to May 2016. Hematological and pediatric phase I were excluded.

**Results:**

A total of 1333 phase I were identified and 15 trials were identified with HRQoL as endpoint (1.1%). The European Organisation for Treatment of Cancer Quality of Life Questionnaire C30 (EORTC QLQ-C30) was the most frequently used instrument: 5 studies (33.3%). The targeted dimensions of HRQoL and the minimal clinically important difference were prespecified in 1 study (6.7%) and 2 studies (13.3%), respectively. Twelve studies (80%) described the statistical approach to analyze HRQoL data. Eight studies used the mean change from baseline (60%) to analyse longitudinal HRQoL data, two the mean score at certain times (13.3%), one the linear mixed model for repeated measures (6.7%), one the time to HRQoL score deterioration (6.7%), one percentage of patient-reported symptoms (6.7%). None of the studies used HRQoL to determine the recommended doses.

**Conclusion:**

Few phase I studies used HRQoL as endpoint and among studies with HRQoL as endpoint, the methodology of HRQoL measurement and statistical analysis was heterogeneous. HRQoL.

endpoint not used for assessing the recommended phase II doses.

## Background

Phase I trials aim to identify the recommended dose for further development of novel drugs under investigation, or recommended phase II Dose (RP2D). This is a key point for the development of new therapeutic strategies the efficacy and toxicity observed in phase II trials and therefore the development of the agent depends on the accuracy of this dose. The classic paradigm that the optimal RP2D has to be the maximal tolerated dose (MTD) has been challenged within the last years, especially concerning molecularly targeted agent. Opinions diverge also on what type and grades of toxicities should be used to define dose-limiting toxicity (DLT) [1, 2]. The DLT is most of the time determined according to the National Cancer Institute Common Terminology Criteria for Adverse Events (NCI-CTCAE) targeting grades 3 and 4.

Doses recommended based on current MTD definition could be higher than needed especially for molecularly targeted agents, carrying the risk of unnecessary toxicities [3].

The duration of toxicity and/or the occurrence of late toxicity are not taken into account in the definition of DLT [3]. Moderate toxicities experienced over a long period could impair patients’ HRQoL and are not taken into account in the usual definition of DLT based on the NCI-CTCAE assessed by clinicians [4]. Moreover, the accuracy of physicians reporting of chemotherapy adverse events have proven weak sensitivity and specificity when compared to patients reported outcomes (PROs) [5]. Therefore, the usual definition of DLT may not appropriately reflect the patient’s feelings regarding the tolerability of the received treatment.

HRQoL and/or PROs could be a complement to the NCI-CTCAE scale to detect adverse events and define the doses.

The objective of this study was to assess the current use of HRQoL as an endpoint (primary or secondary) in phase I oncologic trials. However, its use is most likely not widely spread, as primary or secondary endpoint.

## Methods

### Search strategy and selection for studies

Eligible trials were phase I trials in oncology with HRQoL as endpoint (primary or secondary). Literature searches in PubMed database (January 2012 to May 2016) were performed. Literature search was performed from 2012 to be representative of the current use of HRQoL in phase I trials. We focused on studies in adults solid tumors. Therefore, haematological and pediatric phase I were excluded.

The following strategies were used: (Neoplasms[MeSH Terms] OR neoplasm*[Title/Abstract] OR cancer*[Title/Abstract]) AND (“clinical trial, phase i”“[Publication Type]” OR (trial*[Title/Abstract] AND (phase I[Title/Abstract] OR phase 1[Title/Abstract] OR phase one [Title/Abstract]))).

### Data extraction

Two authors (F. F., A.O) independently extracted information using predefined data abstraction forms. All data were checked for internal consistency, and disagreements were resolved by discussion among the investigators. The following data were extracted: general items (number of patients, year of publication, study period, number of centers, nationality of the first author, academic, mixed or industrial trial), primary endpoint, design of the study, items related to HRQoL measurement, statistical analysis and reporting (rational for HRQoL assessment, methods of data collection, HRQoL questionnaire, evidence of HRQoL questionnaire validity, method/algorithm for scoring the questionnaire, planned schedule of questionnaires, definition of the minimal clinically important difference (MCID), methods used to analyse longitudinal HRQoL data, etc).

### Data analyses

We conducted a descriptive analysis of relevant publications. Quantitative variables were described by median and range. Qualitative variables were described by absolute frequencies (number) and relative frequencies (proportion).

Analyses were conducted with the use of SAS software, version 9.3 (SAS Institute).

## Results

### Characteristics of the studies

**Fig. 1:**
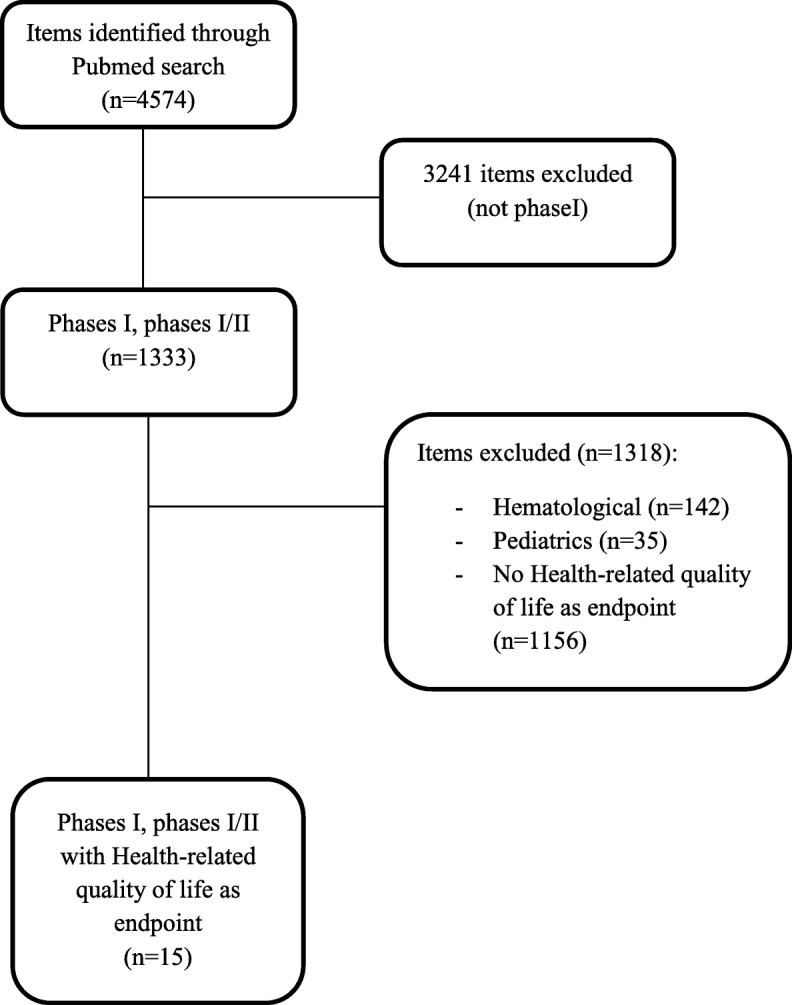
Flow-chart

The characteristics of the studies are described in Table 1. A total of 1333 phase I articles were identified and 15 trials were identified with HRQoL as endpoint (1.1%) (Fig. 1). Among these 15 trials, 14 (93.3%) were academic studies and 1 (6.7%) had a mixed financial support. Twelve studies (80%) enrolled patients with advanced cancers and 3 studies (20%) with localized cancers. The trial concerned targeted therapy (2 trials, 13.3%), chemotherapy (2, 13.3%), immunotherapy (2, 13.3%), radiotherapy associated with a targeted therapy (2, 13.3%), radiotherapy (1, 6.7%), surgery (1, 6.7%), chemoembolization (1, 6.7%) or others (4, 26.6%). Regarding the design, 8 studies (53.3%) used a modified Fibonacci dose escalation based on a 3 + 3 scheme, 2 (13.3%) used a continual reassessment method and 5 (33.3%) other methods. MTD was the most frequent primary endpoint (60%).

**Table 1:** Characteristics of the phase I trials

First author	Patients	Treatment	Design	Primary endpoint	Secondary endpoint
Ringash [8]	Locally advanced head and neck cancer	Radiotherapy	Fleming multi-step algorithm dose escalation	incidence of radiation-induced acute grade 4 toxicity	
Crew [14]	Localized breast cancer	Polyphenon E	Time-to-event continual reassessment method	MTD	Dose-related biologic effects, HRQoL
Xiao [15]	Advanced hepatocellular carcinoma	Tyroserleutide	3 + 3 dose escalation design	MTD, 6-month OS, response, HRQoL	
Lin [16]	Advanced breast cancer	Molecular targeted therapy + radiotherapy	3 + 3 dose escalation design	MTD	ORR, non CNS ORR, PFS, OS, site of first progression, progression free at 6 months, cause of deaths, HRQoL
Kanai [17]	Advanced pancreatic and biliary tract cancer	Curcumin	Rolling 6	MTD, safety, pharmacokinetic, response rate, HRQoL	
Tsubata [9]	Advanced non-small cell lung cancer	Chemotherapy	Not clear	MTD	HRQoL, pharmacokinetic
Han [18]	Localized colorectal cancer	Calcium-magnesium	Randomized, double-blind, placebo controlled	Pharmacokinetic	EMG, patient-reported neurotoxicity symptoms, study treatment preference for reducing neurotoxicity
Rouanne [19]	Advanced cancers	Molecular targeted therapy	NA	HRQoL (ancillary study)	
Stephenson [20]	Advanced cancers	Ascorbic acid	3 + 3 dose escalation design	MTD, pharmacokinetic, HRQoL	
Hunn [21]	Glioblastoma	Vaccine	Single cohort	Feasibility, safety	Response rate, 6 month PFS, rate of vaccine-induced immune response, HRQoL
Reiss [22]	Advanced cancer with peritoneal carcinomatosis	Molecular targeted therapy + radiotherapy	3 + 3 dose escalation design	Safety	Response rate, HRQoL
Cusi [23]	Advanced cancers	Vaccine	3 + 3 dose escalation design	MTD, most effective biological dose	Response, PFS, OS, HRQoL
McRee [24]	Advanced cancers	Molecular targeted therapy	3 + 3 dose escalation design	MTD	Safety, response, HRQoL
Chin [25]	Localized prostate cancer	Surgery	Single cohort	Safety, feasibility	response, HRQoL
Anota [11]	Advanced hepatocellular carcinoma	Chemoembolisation	Continual reassessment method	MTD	HRQoL

*HRQoL* Health-related quality of life, *MTD* maximum tolerated dose, *OS* overall survival, *PFS* progression free survival, *ORR* overall response rate, *CNS* central nervous system

### HRQoL measurement

HRQoL was not used as primary endpoint in any trials (one ancillary study). Two trials (13.3%) provided no result for HRQoL. The EORTC Quality of Life Questionnaire C30 (QLQ-C30) was the most frequently used instrument (5 studies (33.3%)). The reference of the HRQoL instrument validation was provided in 6 studies (40%). The planned schedule of HRQoL assessment was reported in 10 trials (66.7%) (Table 2).

**Table 2:** Aspects relevant to HRQoL statistical analysis

First author	HRQoL questionnaire	Timing of assessment	Targeted dimensions	MCID	Statistical approach for HRQoL analysis	QoL data interpretation
Ringash [8]	FACT-HN	Baseline, 6 and 12 months after completion of radiotherapy			Mean change at 6 months, mean change at 12 months, linear mixed model for repeated measure	Better through time in all groups and no relationship with dose level of radiation
Crew [14]	SF-36	Baseline and 6 months				Finally, no significant change in quality of life as assessed by the SF-36 was observed in either treatment group
Xiao [15]					Mean change from baseline	Not reported
Lin [16]	FACT-B	Baseline, 6 and 12 months			Mean change from baseline	No comparison between the different tested doses (data missing because of death or withdrawal. « Quality of life according to the FACT-Br was generally worse at 6 months for women on study. However, the interpretations of these data are limited by the small number of women with results at both time points and because over half of patients had progressed by that time. »
Kanai [17]	QLQ-C30	Each cycle			Baseline vs best score during treatment intake	No comparison between the dose groups. « Since improved QOL has been demonstrated to contribute to a better outcome in cancer patients it is tempting to speculate that Theracurmin® can improve the outcome of cancer patients through an improvement in QOL. »
Tsubata [9]	FACT-L	Every 4 weeks until 12 weeks after the start of treatment			Mean score at 12 weeks	No differences observed between the treatment doses. « The overall response rate was 1/8, toxicity was acceptable and the QOL score was good. This regimen is therefore suitable for use in elderly patients with NSCLC in an outpatient setting. »
Han [18]		After treatment cycles 1 and 2			Percentage	No treatment effect. « Significantly fewer patients preferred Ca/Mg infusions for reducing their neurotoxicity symptoms than those who preferred placebo or neither treatment (26% versus 74%; *P* = 0.01). »
Rouanne [19]	SF-12, BDI-SF, FSFI, IIEF	Baseline, 1 month			Mean change from baseline	« The patients scored statistically significant decrease in physical health component on the SF-12 after 1-month treatment whereas mental component scale scores did not differ significantly. »
Stephenson [20]	QLQ-C30	Baseline and prior each infusion of vitamin C			Mean score at each cycle	Not used in the discussion.
Hunn [21]	QLQ-C30					Not used in the discussion.
Reiss [22]	QLQ-C30	Baseline and every 2 cycles		10 points	Mean change from baseline	Extensive interpretation. « Our regimen was well tolerated by measures of toxicity and by our QoL data. » « Those who discontinued therapy early, either due to progression of disease, adverse effects, or a global inability to tolerate further treatment, may have had inferior QoL while on this regimen as compared with those who were able to continue the regimen. «
Cusi [23]					Mean change from baseline	Not used in the discussion.
McRee [24]	PHQ-9, GAD-7					Not reported
Chin [25]	IPSS, IIEF-15, UCLA-PCI-SF				Mean change from baseline	« Functional outcomes, IPSS and IIEF-15, both showed a favorable anticipated trend of initial deterioration with subsequent gradual improvement toward baseline levels. »
Anota [11]	QLQ-C30	Baseline and at days 15, 30 and 60 after chemoembolisation	Global health status, physical functioning, fatigue and pain	5 points	Time to deterioration	Overall, patients included at the 10 mg idarubicin dose level seemed to have a longer TTD of HRQoL than patients included at the 5 mg or 15 mg idarubicin dose levels. HRQoL results are thus consistent with the 10 mg idarubicin dose level selected as the MTD

*FACT-HN* Functional Assessment of Cancer Therapy Head and neck, *SF-36* Short form of the medical outcomes study, *FACT-B* Functional Assessment of Cancer Therapy Breast, *QLQ-C30* European Organization for Research and Treatment of Cancer Core Quality of life Questionnaire, *FACT-L* Functional Assessment of Cancer Therapy Lung, *SF-12* Short Form-12 Health, *BDI-SF* Beck Depression Inventory Short-Form, *FSFI* Female Sexual Function Index, *IIEF* International Index of Erectile Function, *PHQ-9* Patient Health Questionnaire, *GAD-7* Generalized Anxiety Disorder 7-item, *IPSS* International Prostate Symptom Score, *UCLA-PCI-SF* Bowel habits domain of University of California, Los Angeles Prostate Cancer Index-Short from

### Statistical analysis of HRQoL

The targeted dimensions of HRQoL were prespecified in one study (6.7%). Two studies (13.3%) determined the minimal clinically important difference (MCID). These two studies used the EORTC QLQ-C30 questionnaire, one used a 5-point difference and one 10-point difference in the HRQoL scores as the MCID. Three studies (20%) mentioned the population data set for HRQoL analysis and all of them used a modified intention-to-treat analysis.

The number of HRQoL data at baseline and at subsequent time points, the HRQoL scores at baseline for each group and each dimension, the profile of missing data at baseline, the statistical approaches for dealing with missing data were adequately reported in 3 (20%), 2 (13.3%), 3 (20%) and 1 (6.7%) studies, respectively. The method for dealing with missing data was the pattern mixture method. No study provided the reasons why data were missing. Twelve studies (80%) described the statistical approach to analyze HRQoL data. The different statistical methods/analyses were: the mean change from baseline for 8 studies (60%), mean score at certain times for two studies (13.3%), linear mixed model for repeated measures (LMM) (one study, 6.7%), time to HRQoL score deterioration (TTD) (1 study, 6.7%), percentage of patient-reported symptoms (1, 6.7%). None of the studies identified the RP2D using HRQoL measurement (Table 2).

## Discussion

To our knowledge, this is the first systematic review related to the use of HRQoL in phase I oncology trials. We demonstrated that few phase I studies used HRQoL as endpoint showing that HRQoL is not yet considered of major interest in phase I trials. Moreover, among studies with HRQoL as endpoint, none identified the RP2D according to the HRQoL results and the methodology of HRQoL measurement and statistical analysis was heterogeneous.

The MTD is determined by DLT which is usually defined as any grade 3–4 non-haematological or grade 4 haematological toxicity occurring during the first cycle of treatment. Nevertheless, Paoletti et al., in an international survey implying 65 experts, showed that these experts are willing to consider some grade 2 toxicities as DLT and that the notion of evolution of the symptoms should be regarded as important while it is poorly assessed in current practice [6]. These moderate toxicities are not taken into account in the usual definition of DLT based on the NCI-CTCAE assessed by clinicians at cycle 1. Therefore, the usual definition of DLT may not appropriately reflect the patient’s feelings regarding the tolerability of the treatment received. Some moderate grade 2 adverse events may have an impact on quality of life and in consequence on compliance of the treatment.

Moreover, 50% of the grade 3/4 toxicities of molecular targeted therapies seem to occur after cycle 1. Postel-Vinay et al. presented data showing that, among a large number of patients participating in molecular targeted therapies phase I trials, response rate is not confined to patients treated at doses close to the MTD [3].

Furthemore, Henon and all showed in a survey including 52 patients enrolled in 27 phase I trials that the patients’ most feared AEs are gastrointestinal toxicities, neurological toxicities and personality change, which differs from the physicians’ most feared toxicities [7]. We also know that there may be discrepancies between patients and physicians reporting toxicities.

In that context, HRQol and patient-reported outcomes may be an added value as a complement to the usual NCI-CTCAE scale to detect toxicities and define the RP2D. Nevertheless, this review shows that HRQoL has been poorly investigated in oncology phase I clinical trials. None of the studies included in our review used HRQoL as primary endpoint or identified the RP2D using HRQoL measurements. Three studies analyzed HRQoL according to the dose levels. In the Ringash et al. [8] and Tsubata et al. [9] studies there was no relationship between dose level and HRQoL while they observed increased toxicities according to the dose of treatment. In Anota et al. study, patients presented a longer TTD at the MTD (10 mg idarubicin) than at the lower level (5 mg idarubicin) for global health status, physical functioning, fatigue and pain dimensions. These results consolidate the selection of the RP2D.

In the perspective of enabling patients to speak on themselves, without the filter of physicians, a patient-reported outcomes version of the common terminology criteria for adverse events (PRO-CTCAE) was developed by the National Cancer Institute [10]**.** These PRO-CTCAEs could be a complement of the NCI-CTCAEs to define the DLT or we could use the PRO-CTCAEs scale rather than the NCI-CTCAEs scale to determine the RP2D in a more patient-oriented perspective. Nevertheless, some moderate adverse events which could have an impact on patients’ HRQoL over time might not be taken into account by the PRO-CTCAE. Further researches are mandatory and the first step could be to implement PRO-CTCAEs as secondary endpoint in order to compare the results obtained with the NCI-CTCAEs.

HRQoL questionnaires could bring an added value in phase I as a complement to results obtained on toxicity, as the study of Anota et al. [11] to see if there’s no impact on HRQoL at the MTD compared to lower doses. A longitudinal analysis of HRQoL could be an alternative way to assess the impact of the MTD in a clinically meaningful way. The two main methods for longitudinal analysis of HRQOL data in oncology are to be considered: the linear mixed model for repeated measures or the time to HRQoL score deterioration [12]. Nevertheless, no guidelines exist for the MCID definition and the longitudinal analysis of HRQoL. The optimal definition of MCID should be explored according to cancer localization, treatment, and setting with and evidence-based approach and guidelines. Ongoing SISAQOL and qRECIST phase III projects, supported by the EORTC, are warranted for longitudinal HRQOL [13]. Once these guidelines for phase III trials will be published, methodological researches will be mandatory to analyze their potential implementation in phase I trials.

The main limitation of our study is the short number of studies with HRQoL as endpoints. None of them identified the RP2D with HRQoL as endpoint. Therefore, we are unable to provide any example of a drug that is used in the daily life and which dose has been determined by HRQoL or PROs in phase I. Moreover, the side-effects of the drugs are frequently not known as many drugs assessed in phase I are first-in-human and can be first-in-kind. Therefore, the choice of a HRQoL instruments can be difficult in this context.

## Conclusion

HRQoL could be an added value for the assessment of the RP2D and further methodological research is necessary to implement HRQoL in oncology phase I trials.

### Aknowledgements

Not applicable.

## Abbreviations

DLT: Dose-limiting toxicity
EORTC QLQ-C30: European Organisation for Treatment of Cancer Quality of Life Questionnaire C30
HRQoL: Health-related quality of life
MCID: Minimal clinically important difference
MTD: Maximal tolerated dose
NCI-CTCAE: National Cancer Institute Common Terminology Criteria for Adverse Events
PRO-CTCAE: Patient-reported outcomes version of the common terminology criteria for adverse events
PROs: Patients reported outcomes
RP2D: Recommended phase II Dose
TTD: Time to HRQoL score deterioration

## References

[1] Haines IE (2008). Dose selection in phase I studies: why we should always go for the Most effective. J Clin Oncol.

[2] Sleijfer S, Wiemer E (2008). Dose selection in phase I studies: why we should always go for the top. J Clin Oncol.

[3] Postel-Vinay S, Gomez-Roca C, Molife LR, Anghan B, Levy A, Judson I (2011). Phase I trials of molecularly targeted agents: should we pay more attention to late toxicities?. J Clin Oncol.

[4] Verweij J, Disis ML, Cannistra SA (2010). Phase I studies of drug combinations. J Clin Oncol.

[5] Fromme EK, Eilers KM, Mori M, Hsieh Y-C, Beer TM (2004). How accurate is clinician reporting of chemotherapy adverse effects? A comparison with patient-reported symptoms from the quality-of-life questionnaire C30. J Clin Oncol.

[6] Paoletti X, Le Tourneau C, Verweij J, Siu LL, Seymour L, Postel-Vinay S (2014). Defining dose-limiting toxicity for phase 1 trials of molecularly targeted agents: results of a DLT-TARGETT international survey. Eur J Cancer.

[7] Henon C, Lissa D, Paoletti X, Thibault C, Le TC, Lanoy E (2017). Patient-reported tolerability of adverse events in phase 1 trials. ESMO Open.

[8] Ringash J, Lockwood G, O’Sullivan B, Warde P, Bayley A, Cummings B (2008). Hyperfractionated, accelerated radiotherapy for locally advanced head and neck cancer: quality of life in a prospective phase I/II trial. Radiother Oncol.

[9] Tsubata Y, Okimoto T, Miura K, Karino F, Iwamoto S, Tada M (2013). Phase I clinical and pharmacokinetic study of bi-weekly carboplatin/paclitaxel chemotherapy in elderly patients with advanced non-small cell lung cancer. Anticancer Res.

[10] Basch E, Reeve BB, Mitchell SA, Clauser SB, Minasian LM, Dueck AC (2014). Development of the National Cancer Institute’s Patient-Reported Outcomes Version of the Common Terminology Criteria for Adverse Events (PRO-CTCAE). JNCI J Natl Cancer Inst.

[11] Anota A, Boulin M, Dabakuyo-Yonli S, Hillon P, Cercueil J-P, Minello A (2016). An explorative study to assess the association between health-related quality of life and the recommended phase II dose in a phase I trial: idarubicin-loaded beads for chemoembolisation of hepatocellular carcinoma. BMJ Open.

[12] Bonnetain F, Fiteni F, Efficace F, Anota A (2016). Statistical challenges in the analysis of health-related quality of life in Cancer clinical trials. J Clin Oncol.

[13] Bottomley A, Pe M, Sloan J, Basch E, Bonnetain F, Calvert M (2016). Analysing data from patient-reported outcome and quality of life endpoints for cancer clinical trials: a start in setting international standards. Lancet Oncol.

[14] Crew KD, Brown P, Greenlee H, Bevers TB, Arun B, Hudis C (2012). Phase IB randomized, double-blinded, placebo-controlled, dose escalation study of polyphenon E in women with hormone receptor-negative breast cancer. Cancer Prev Res.

[15] Xiao Z-Y, Jia J-B, Chen L, Zou W, Chen X-P (2012). Phase I clinical trial of continuous infusion of tyroserleutide in patients with advanced hepatocellular carcinoma. Med Oncol.

[16] Lin NU, Freedman RA, Ramakrishna N, Younger J, Storniolo AM, Bellon JR (2013). A phase i study of lapatinib with whole brain radiotherapy in patients with human epidermal growth factor receptor 2 (HER2)-positive breast cancer brain metastases. Breast Cancer Res Treat.

[17] Kanai M, Otsuka Y, Otsuka K, Sato M, Nishimura T, Mori Y (2013). A phase i study investigating the safety and pharmacokinetics of highly bioavailable curcumin (Theracurmin??) in cancer patients. Cancer Chemother Pharmacol.

[18] Han CH, Khwaounjoo P, Kilfoyle DH, Hill A, McKeage MJ (2013). Phase I drug-interaction study of effects of calcium and magnesium infusions on oxaliplatin pharmacokinetics and acute neurotoxicity in colorectal cancer patients. BMC Cancer.

[19] Rouanne M, Massard C, Hollebecque A, Rousseau V, Varga A, Gazzah A (2013). Evaluation of sexuality, health-related quality-of-life and depression in advanced cancer patients: a prospective study in a phase i clinical trial unit of predominantly targeted anticancer drugs. Eur J Cancer.

[20] Stephenson CM, Levin RD, Spector T, Lis CG (2013). Phase i clinical trial to evaluate the safety, tolerability, and pharmacokinetics of high-dose intravenous ascorbic acid in patients with advanced cancer. Cancer Chemother Pharmacol.

[21] Hunn MK, Bauer E, Wood CE, Gasser O, Dzhelali M, Ancelet LR (2015). Dendritic cell vaccination combined with temozolomide retreatment: results of a phase I trial in patients with recurrent glioblastoma multiforme. J Neuro-Oncol.

[22] Reiss KA, Herman JM, Zahurak M, Brade A, Dawson LA, Scardina A (2015). A phase I study of Veliparib (ABT-888) in combination with low-dose fractionated whole abdominal radiation therapy in patients with advanced solid malignancies and peritoneal Carcinomatosis. Clin Cancer Res.

[23] Cusi MG, Botta C, Pastina P, Rossetti MG, Dreassi E, Guidelli GM (2015). Phase I trial of thymidylate synthase poly-epitope peptide (TSPP) vaccine in advanced cancer patients. Cancer Immunol Immunother.

[24] McRee AJ, Sanoff HK, Carlson C, Ivanova A, O’Neil BH (2015). A phase i trial of mFOLFOX6 combined with the oral PI3K inhibitor BKM120 in patients with advanced refractory solid tumors. Investig New Drugs.

[25] Chin JL, Billia M, Relle J, Roethke MC, Popeneciu IV, Kuru TH (2016). Magnetic resonance imaging–guided transurethral ultrasound ablation of prostate tissue in patients with localized prostate Cancer: a prospective phase 1 clinical trial. Eur Urol.

